# Coordination of Protein Kinase and Phosphoprotein Phosphatase Activities in Mitosis

**DOI:** 10.3389/fcell.2018.00030

**Published:** 2018-03-22

**Authors:** Isha Nasa, Arminja N. Kettenbach

**Affiliations:** ^1^Department of Biochemistry and Cell Biology, Geisel School of Medicine at Dartmouth, Hanover, NH, United States; ^2^Norris Cotton Cancer Center, Geisel School of Medicine at Dartmouth, Lebanon, NH, United States

**Keywords:** phosphatases, kinases and phosphatase, mitosis, protoemics, mass spectrometry

## Abstract

Dynamic changes in protein phosphorylation govern the transitions between different phases of the cell division cycle. A “tug of war” between highly conserved protein kinases and the family of phosphoprotein phosphatases (PPP) establishes the phosphorylation state of proteins, which controls their function. More than three-quarters of all proteins are phosphorylated at one or more sites in human cells, with the highest occupancy of phosphorylation sites seen in mitosis. Spatial and temporal regulation of opposing kinase and PPP activities is crucial for accurate execution of the mitotic program. The role of mitotic kinases has been the focus of many studies, while the contribution of PPPs was for a long time underappreciated and is just emerging. Misconceptions regarding the specificity and activity of protein phosphatases led to the belief that protein kinases are the primary determinants of mitotic regulation, leaving PPPs out of the limelight. Recent studies have shown that protein phosphatases are specific and selective enzymes, and that their activity is tightly regulated. In this review, we discuss the emerging roles of PPPs in mitosis and their regulation of and by mitotic kinases, as well as mechanisms that determine PPP substrate recognition and specificity.

## Introduction

Transitions between distinct phases of the cell cycle are governed by post-translational modifications of proteins. Protein phosphorylation is the most prevalent post-translational modification, with more than three-quarters of the human proteome being phosphorylated (Sharma et al., 2014). The highest occupancy of phosphorylation sites is observed in mitosis, where transcription and translation are repressed. Entry into, progression through, and exit from mitosis are tightly regulated by the highly dynamic phosphorylation of cell cycle-specific proteins.

Entry into mitosis is characterized by a peak in protein phosphorylation. This increase in protein phosphorylation is not only the consequence of increased kinase activity but is accompanied by a concurrent decrease in phosphoprotein phosphatase activity. Indeed, inhibition of phosphoprotein phosphatase activities in interphase cells is sufficient to induce a pseudo-mitotic state characterized by an increase in Cyclin-dependent kinase 1 (Cdk1) activity, chromosome condensation, and microtubule aster formation (Yamashita et al., 1990; Gowdy et al., 1998). Cdk1 is the major force for protein phosphorylation in mitosis and its activation triggers a switch-like and irreversible transition from interphase into mitosis. Pioneering work from genetic analyses of budding and fission yeasts and biochemical analyses in frog and clam egg extracts showed that cyclins form a stoichiometric complex with Cdk1, activate it, and drive cell cycle transitions (Masui and Markert, 1971; Rosenthal et al., 1980; Gerhart et al., 1984; Morgan, 1995; Nurse et al., 1998). Furthermore, once activated, Cdk1 controls phosphorylation-dependent feedback loops involving kinases and phosphatases generating a bistable switch which promotes transition from interphase to mitosis and protecting the cell from either premature entry into mitosis or slippage back into interphase (Morgan, 2007; Salaun et al., 2008). Once activated, Cdk1 with its partner cyclin B, directly and indirectly, regulates most mitotic phosphorylation events (Salaun et al., 2008; Wurzenberger and Gerlich, 2011; Qian et al., 2013b). Analyses of Cdk1 substrates in different yeast strains and in human cells have identified several hundred proteins that are phosphorylated by Cdk1 in mitosis (Ubersax et al., 2003; Blethrow et al., 2008; Holt et al., 2009; Petrone et al., 2016; Swaffer et al., 2016). In addition, mitotic kinases including Polo like kinase 1 (Plk1), Aurora A/B (AURKA/B), Greatwall kinase (Gwl), Wee1, Mps1, Haspin, and NIMA-related kinases are activated in mitosis and contribute to the marked increase in protein phosphorylation in mitosis (Nigg, 2001; O'Farrell, 2001; Kettenbach et al., 2011; Oppermann et al., 2012; Maiolica et al., 2014; Cundell et al., 2016; Lera et al., 2016; Cullati et al., 2017; Maciejowski et al., 2017).

Exit from mitosis and re-establishment of lower interphase phosphorylation levels is accomplished by degradation of mitotic phosphoproteins and reversal of mitotic phosphorylation. Currently, it is estimated that approximately 170 human proteins are being degraded during mitotic exit (Min et al., 2014). Protein degradation is essential for mitotic exit, as it ensures uni-directionality, and reduces the activities of mitotic kinases such as Cdk1/cyclin B, AURKA/B, and Plk1 (Draetta et al., 1989; King et al., 1995; Littlepage and Ruderman, 2002; Lindon and Pines, 2004; Stewart and Fang, 2005). However, protein degradation and consequently the decline of mitotic kinase activities is not sufficient to trigger mitotic exit (Queralt and Uhlmann, 2008). In addition, to ensure safe passage through and exit from mitosis, as well as reinstatement of interphase phosphorylation levels, ordered and controlled dephosphorylation of mitotic phosphoproteins is essential.

Here, we review recent findings on the roles of phosphoprotein phosphatases as specific and selective regulators of mitosis, their different mechanisms of substrate recognition, and their inter- and counter-actions with mitotic kinases.

## Protein phosphatases

Protein phosphatases have been classified into four major classes based on their substrate preference, inhibitor sensitivity and catalytic mechanism. These classes include phosphoprotein phosphatases (PPP), Mg^2+^/Mn^2+^-dependent protein phosphatases (PPM), phosphotyrosine phosphatases (PTP), and aspartate-based protein phosphatases (Kerk et al., 2008). There are 189 known and predicted protein phosphatase genes and 539 protein kinase genes encoded in the human genome (Chen et al., 2017). While the majority of protein kinases (~400) specifically phosphorylate serine and threonine amino acids, only ~30 protein phosphatases are serine and threonine specific. Furthermore, the majority of cellular serine/threonine dephosphorylation has been attributed to two members of the PPP family: Protein Phosphatase 1 (PP1) and 2A (PP2A) (Moorhead et al., 2007; Virshup and Shenolikar, 2009; Bollen et al., 2010). This imbalance in the number of protein kinases and phosphatases as well as the observation that protein kinases, but not PPPs, exhibit site specificity *in vitro*, led to the belief that PPPs are unspecific, constitutively active “housekeeping” enzymes while protein kinases are the primary determinants of phosphorylation signaling (Brautigan, 2013). However, it has recently become clear that protein phosphatases are specific, selective, and tightly regulated enzymes. For the PPP family, specificity, distinct cellular localization, and regulation is achieved when catalytic subunits associate with non-catalytic subunits to form multimeric holoenzymes. Each of these holoenzymes functions as a distinct signaling entity by modulating the activity of PPP catalytic subunits and establishing their substrate specificity. Combinatorially, PPPs are efficient holoenzymes, and expand the number of functional phosphatases to several hundred by associating with partner regulatory proteins.

## Phosphoprotein phosphatases (PPP)–multimeric holoenzymes

The PPP family of protein phosphatases consists of PP1, PP2A, PP2B (also known as calcineurin or PP3), PP4, PP5, PP6, and PP7 (Table 1). All members of the PPP family are defined by three highly conserved signature sequence motifs (GDXHG-, -GDXVDRG-, and -GNHE-), which establish the catalytic active site (Cohen, 2002; Wang et al., 2008). These amino acids coordinate two divalent metal ions (either Mn^2+^, Fe^2+^, and Zn^2+^ ions) in the catalytic center. The metal ions in the catalytic center of PPPs are crucial for the activation of a water molecule which triggers a nucleophilic attack on the phosphorous atom of the substrate phosphate group to hydrolyze the phosphate ester bond, thereby dephosphorylating the substrate (Barford et al., 1998).

**Table 1:** Human phosphoprotein phosphatase (PPP) catalytic, regulatory, and scaffolding subunit genes and their isoforms.

**PPP catalytic subunit genes**		**Regulatory genes**		**Scaffold genes**	
**Family**	**Isoforms**	**Family**	**Isoforms**	**Family**	**Isoforms**
Protein phosphatase 1 (PPP1C)	PPP1CA PPP1CB PPP1CG	>200		None	
Protein phosphatase 2A (PPP2C)	PPP2CA PPP2CB	B (B55) B′ (B56) B″ (B72) B^‴^ (Striatin)	PPP2R2A (B55α) PPP2R2B (B55β) PPP2R2C (B55γ) PPP2R2D (B55δ) PPP2R5A (B56α) PPP2R5B (B56β) PPP2R5C (B56γ) PPP2R5D (B56δ) PPP2R5E (B56ε) PPP2R3A (PR72) PPP2R3B (PR70) PPP2R3C (GSPR) PPP2R3D (PR59) STRN (Striatin) STRN3 (SG2NA) STRN4	A (PR65)	PPP2R1A PPP2R1B
Protein phosphatase 2B (PPP3C)			None		
	PPP3CA PPP3CB PPP3CC	B	PPP3R1 PPP3R2		
Protein phosphatase 4 (PPP4C)		PPP4R1 PPP4R2 PPP4R3A PPP4R3B PPP4R4			
Protein phosphatase 5 (PPP5C)					
Protein phosphatase 6 (PPP6C)		PPP6R1 PPP6R2 PPP6R3 ANKRD28 ANKRD44 ANKRD52			
Protein phosphatase 7 (PPP7C)					

Among the PPP family members, we will focus on PP1, PP2A, PP4, and PP6, and their roles in the regulation of mitosis in mammalian cells. There are four PP1 genes in the human genome, which encode the four isoforms of the catalytic subunit: PP1α, PP1β, PP1γ1, and PP1γ2. The four PP1 isoforms share high sequence identity (>90%), but differ in tissue expression and have specific functions by associating with isoform-specific partners (Uhlen et al., 2015). PP1 achieves substrate specificity through the formation of heterodimers consisting of the catalytic subunit with diverse set of about 200 regulatory proteins (Bollen et al., 2010). Within the PPP family, PP2A, PP4, and PP6 catalytic subunits share the highest degree of sequence identity ranging from 60 to 65% and are classified as the PP2A-like subfamily of PPPs. PP2A forms heterotrimers consisting of a catalytic subunit, a scaffolding A subunit, and a regulatory B subunit. The PP2A catalytic subunit has two isoforms (PP2ACα and PP2ACβ), which share 97% sequence identity with each other. The PP2A scaffolding subunit A also has two isoforms (PP2AAα and PP2AAβ), which share about 87% sequence identity with each other. The catalytic and scaffolding subunit assemble into a core dimer which is joined by a regulatory B subunit to form the heterotrimeric PP2A holoenzyme. In the human genome there are 16 genes encoding regulatory B subunits. The B subunits are classified into four subfamilies: B55 (PR55/B), B56 (PR61/B′), B72 (PR72/B″), and Striatin (PR93/B^‴^) (Table 1; Seshacharyulu et al., 2013). Theoretically, combinations of these subunits can generate ~100 different PP2A holoenzymes, each with potentially distinct substrate specificity. In mitosis, the B55 and B56 subfamily of PP2A regulatory proteins play the most prominent role and dictate the localization and activity of the PP2A holoenzyme (Foley et al., 2011; Funabiki and Wynne, 2013). PP4 and PP6, the other two members of the PP2A subfamily can form heterodimers or heterotrimers. For PP4, the catalytic subunit PP4C binds either the regulatory subunits PP4R1 or PP4R4 resulting in heterodimers, or PP4R2 and PP4R3α/β to form heterotrimers (Table 1). In human cells, PP6 exists in heterotrimeric form. The PP6 catalytic subunit, PP6C, interacts with one of three regulatory ankyrin-repeat containing proteins, ANR28, ANR44 and ANR52, as well as one of three highly conserved regulatory SAPS (Sit4-Associated Proteins) domain containing proteins, PP6R1, PP6R2 and PP6R3 (Table 1). In yeast, PP6 forms heterodimers with the catalytic subunit only bound to one SAPS-domain containing regulatory subunit homologs. This difference introduces an intriguing dichotomy into the structure and function of PP6 that does not exist for other PPP members and requires further exploration.

## Mitosis entry and exit–more than “bulk” phosphorylation and “bulk” dephosphorylation

**Figure 1:**
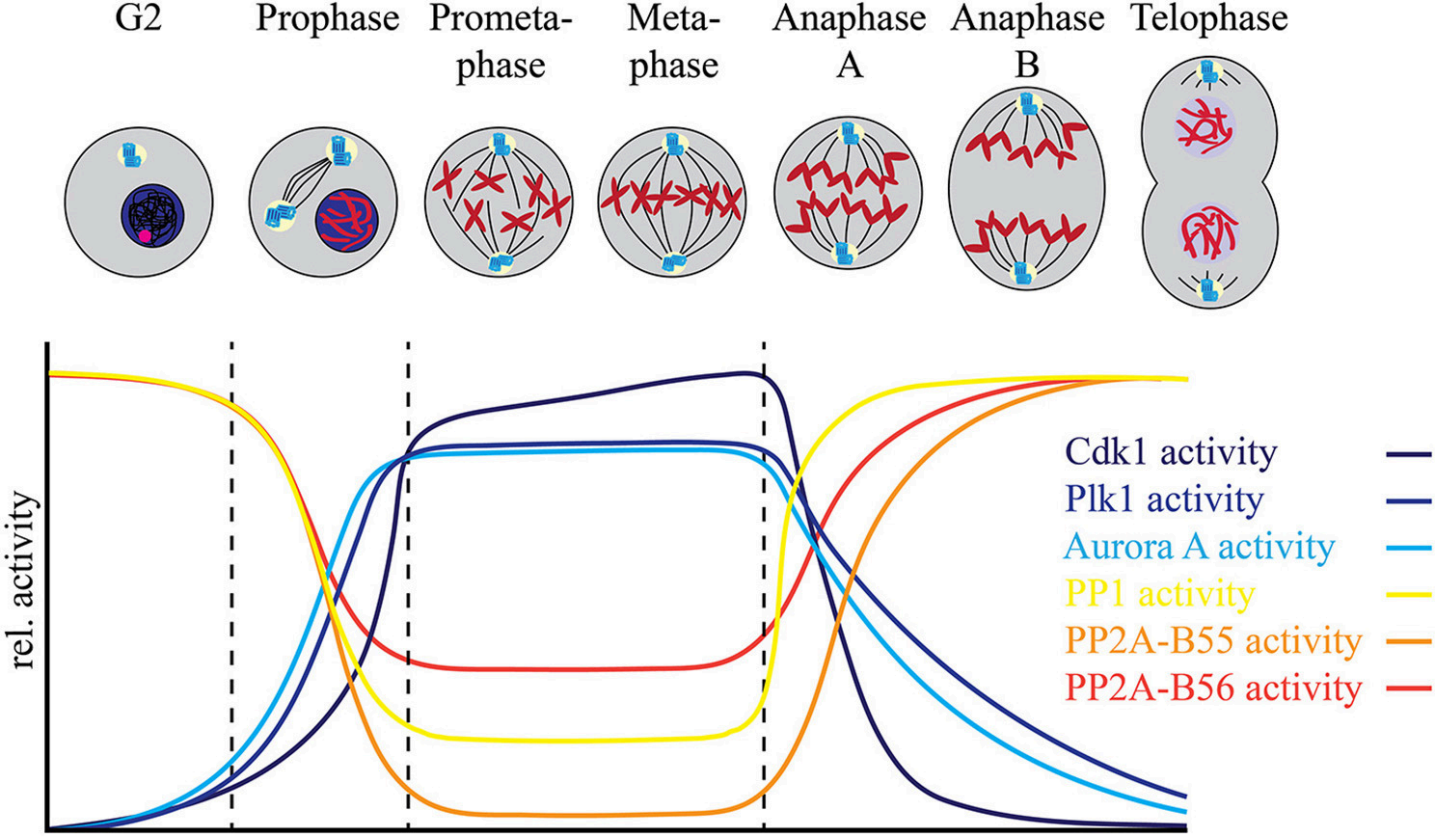
Fine tuning between the mitotic protein kinases and protein phosphatases regulates mitotic progression. The relative activities of major mitotic protein kinases including Cdk1, AURKA, AURKB, and Plk1, indicated in blue spectrum, increase as the cells enter mitosis. This increase is accompanied by a relative decrease in the activities of major mitotic phosphatases including PP1, PP2A-B56 and PP2A-B55. While PP2A-B55 activity is completely inhibited by binding of its inhibitors ENSA and ARPP-19 at mitotic entry, PP2A-B56 is still active at localized complexes and regulates mitotic progression. Similarly, most of the PP1 activity is inhibited by Cdk1 dependent phosphorylation of its C-terminal Thr-320 residue at mitotic entry, but localized PP1 complexes remain active during mitosis. PP1 regains complete activity after the degradation of cyclin B and consequent decline of Cdk1 activity at metaphase-anaphase transition and controls the exit of cells from mitosis.

Entry into mitosis is often characterized as a dramatic increase in “bulk” phosphorylation that needs to be reversed by “bulk” dephosphorylation to allow cells to exit mitosis. These changes in protein phosphorylation are in general thought to be accompanied by a rise in kinase activity, most importantly Cdk1 activity, and a reduction in PP1 and PP2A phosphatase activities early in mitosis, followed by a reversal of the respective activities as cells start to exit mitosis. However, regulation of phosphorylation signaling in all phases of mitosis is highly dynamic requiring coordination of opposing kinase and PPP activities in a specific temporal and spatial manner to ensure orderly and accurate progression through mitosis to generate identical daughter cells (Figure 1).

Evidence for extensive regulation of PPP activity during mitosis comes from recent advances in mass spectrometry-based proteomics that have enabled the global analysis of the phosphoproteome (Beausoleil et al., 2004; Zhang et al., 2005; Cantin et al., 2006; Kruger et al., 2008; Holt et al., 2009; Olsen et al., 2010; Swaffer et al., 2016). In combination with small molecule kinase inhibitors, these studies have revealed many kinase-substrate relationships and provided insights into complex phosphorylation signaling pathways (Carlson and White, 2011; Kettenbach et al., 2011, 2015; Oppermann et al., 2012; Stuart et al., 2015; Petrone et al., 2016; Maciejowski et al., 2017). A common experimental strategy for these experiments is the quantitative comparison of two cell populations: one treated with kinase inhibitor for a short period of time to avoid changes in protein abundance, and the other control-treated, followed by the comparison of the dynamic changes of phosphorylation site occupancy between them by mass spectrometry. While highly effective, one caveat of this experimental strategy is that upon cessation of kinase activity, the activity of an opposing protein phosphatase is necessary to reduce phosphorylation site occupancy. In other words, in the absence of a counteracting phosphatase, phosphorylation site occupancy will not change even in the presence of inhibitors targeting the responsible kinase. When performed in mitosis or in mitotic arrest induced by microtubule stabilizers such as Taxol or destabilizers such as nocodazole, this strategy has led to the linkage of hundreds to thousands of phosphorylation sites to specific mitotic kinases, suggesting that protein phosphatases are indeed active during mitosis (Figure 1). Too little PPP activity arrests cells in mitosis and prevents their exit, while too much PPP activity results in mitotic defects (Ishida et al., 1992; Burgess et al., 2010; Álvarez-Fernández et al., 2013). This leads to the following questions: How does a cell get the balance of these opposing activities just right? And how are protein kinase and phosphatase activities regulated by each other and on their shared substrates to achieve the balance of phosphorylation?

## Cdk1–the master regulator of mitotic phosphatases?

**Figure 2:**
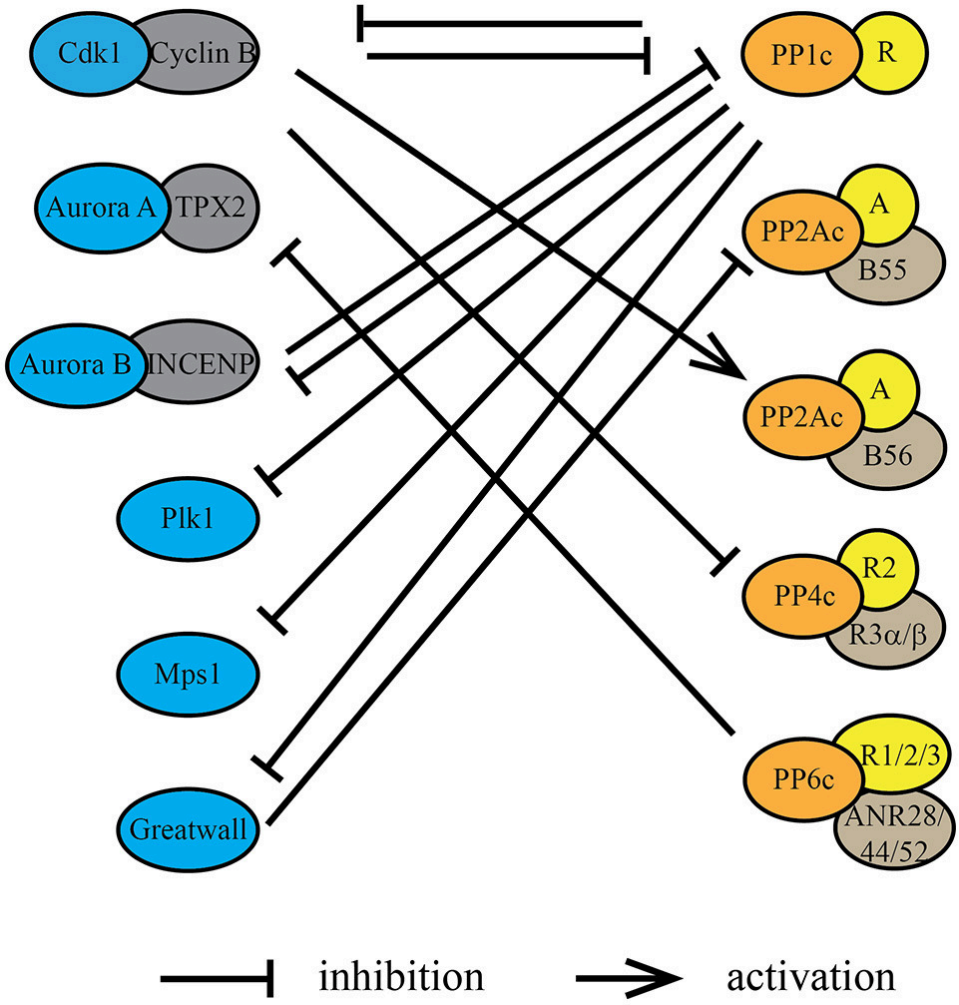
Protein kinases and protein phosphatases regulate each other during mitosis. Protein kinases and protein phosphatases coordinate with each other through underlying dynamic phosphorylation changes within kinase/phosphatase catalytic or regulatory subunits. Cdk1/cyclin B, directly or indirectly, inhibits the phosphatase activity of PP1, PP2A-B55, and PP4. Conversely, PP1 suppresses the kinase activity of AURKB through its regulatory protein Sds22 (PPP1R7), Plk1 through Mypt1 (PPP1R12A) and Gwl through PPP1R3B. Phosphorylation by Cdk1/cyclin B within or near the PP2A-B56 binding LxxIxE motif in substrates increases the affinity of PP2A-B56 interactions. PP2A-B55 activity is inhibited during mitosis by Gwl phosphorylation of the inhibitory proteins ENSA and ARPP-19. PP1 inactivates Gwl at mitotic exit, thereby activating PP2A-B55. PP6 is the T-loop phosphatase for AURKA, thereby decreasing its activity directly.

Entry into mitosis is marked by a stark increase in kinase activity. In late G2, mitotic kinases including Cdk1/cyclin B, Plk1, AURKA, AURKB, and Gwl are activated (Cdk1 activation is in detail described in Morgan, 1995, 2007), resulting in a net increase in substrate phosphorylation (Figure 1). However, this increase in protein kinase activity and consequently substrate phosphorylation is not sufficient to drive entry into mitosis. Along with increased protein kinase activity, the inhibition of specific phosphoprotein phosphatase activities is necessary for a cell to enter mitosis. Timely inhibition of PP2A-B55 by Gwl (discussed in more detail below) is essential for the switch-like and irreversible transition into mitosis (Castilho et al., 2009; Gharbi-Ayachi et al., 2010; Lorca et al., 2010; Mochida et al., 2010; Krasinska et al., 2011). Reduction of PP2A activity in interphase *Xenopus laevis* egg extract was found to be sufficient to trigger premature entry into mitosis at low Cdk activity (Krasinska et al., 2011). Inhibition of PP2A/B55 as well as other members of the PPP family is directly or indirectly controlled by Cdk1/cyclin B itself (Figure 2).

In case of PP1, Cdk1/cyclin B phosphorylates Thr-320 close to the carboxyl-terminus of PP1α/β/γ, which has an inhibitory effect on the catalytic activity of PP1 during mitosis (Dohadwala et al., 1994; Kwon et al., 1997). Phosphoproteomic analyses of protein phosphorylation in a population of nocodazole arrested (“early mitotic”) HeLa cells have determined the occupancy of Thr-320 with a mean of 60% (Olsen et al., 2010), suggesting not all the PP1 in the cell is inactive during mitosis (Figure 1). Reversal of this inhibitory phosphorylation is achieved through auto-catalysis by PP1. A small decrease in Cdk1/cyclin B activity at metaphase-anaphase transition is sufficient for PP1 to auto-dephosphorylate Thr-320 (Wu et al., 2009). Besides the direct post-translational modification of the catalytic subunit, Cdk1/cyclin B also regulates PP1 activity by phosphorylating PP1 regulatory subunits and preventing their binding to the catalytic subunit. This is specifically important for the local regulation of PP1 activity. For instance, Cdk1/cyclin B phosphorylates Repo-Man, preventing PP1 targeting to chromosomes before metaphase-anaphase transition (Vagnarelli et al., 2011). PP1 activity is also modulated through the binding of small, heat stable inhibitory proteins called Inhibitor 1 and 2 (Brautigan, 2013). Phosphorylation of Inhibitor-1 and Inhibitor-2 by PKA (Ceulemans and Bollen, 2004) and Cdk1/cyclin B (Leach et al., 2003), respectively, regulates their binding to the catalytic subunit of PP1. Together these mechanisms account for the reduction, but not complete inhibition of PP1 activity in mitosis.

The Cdk1/cyclin B-dependent inhibition of PP2A-B55 complex is indirect. Upon mitotic entry, Cdk1/cyclin B phosphorylates and activates Gwl kinase, which in turn inhibits PP2A-B55 activity (Castilho et al., 2009; Vigneron et al., 2009). The depletion of Gwl activity in *Xenopus* egg extracts results in dephosphorylation of mitotic phosphoproteins and exit from mitosis, even in the presence of high Cdk1/cyclin B activity, suggesting a crucial role for Gwl in delaying the dephosphorylation of mitotic substrates (Vigneron et al., 2009). Furthermore, addition of okadaic acid, an inhibitor of PP1, PP2A, PP4, PP5, and PP6 phosphatase activities, mitigated the effects of Gwl depletion, supporting the notion that Gwl inhibits a phosphatase activity in cells. Gwl does not directly inhibit PP2A-B55 activity, but phosphorylates two homologous, heat-stable proteins ENSA (α-Endosulfine) and ARPP19 (cyclic adenosine monophosphate–regulated phosphoprotein 19) at a highly conserved serine residue (Mochida et al., 2010; Mochida, 2014). It is the phosphorylated forms of ENSA and ARPP19 that specifically bind and inhibit PP2A-B55 (Lorca and Castro, 2013). This process is regulated in a spatial as well as temporal manner. In G2, Cdk1/cyclin B and PP2A-B55 are localized in the cytoplasm, while Gwl is in the nucleus. In late G2, active Cdk1/cyclin B shuttles into the nucleus, where it phosphorylates Gwl at not only the activation loop resulting in Gwl activation, but also within a nuclear localization sequence resulting in the cytoplasmic translocation of Gwl (Wang et al., 2016). In the cytoplasm, Gwl phosphorylates ENSA and ARPP19, resulting in the inactivation of PP2A-B55, tipping the balance toward a net increase in phosphorylation. Differentially localized populations of active and inactive phosphatase are generated so as to not interrupt the start of Cdk1/cyclin B activation in the nucleus and the spreading of Cdk1/cyclin B activity upon nuclear envelope breakdown (Gavet and Pines, 2010). ENSA and ARPP19 are highly expressed in cells (Sharma et al., 2014). Thus, in late G2 and early mitosis, most PP2A-B55 is in complex with either phosphorylated ENSA or ARPP19, resulting in its strong inhibition. In anaphase, when Cdk1/cyclin B levels and activity decrease and Gwl is inactivated, the pool of phosphorylated ENSA and ARPP19 also decreases, resulting in the reactivation of PP2A-B55 (Figure 1).

Cdk1/cyclin B also directly phosphorylates the protein Bod1. This phosphorylation turns Bod1 into a potent inhibitor of PP2A-B56 complex at kinetochores (Porter et al., 2013). At kinetochores, Bod1 inhibition of PP2A-B56 promotes Plk1 localization and regulates kinetochore-microtubule interactions. Furthermore, phosphorylation of its substrates by Cdk1/cyclin B has been shown to affect the affinity of PP2A-B56 toward these substrates. The effects of Cdk1/cyclin B phosphorylation on PP2A-B56 substrate recognition are discussed in the next section.

Finally, Cdk1/cyclin B phosphorylates the heterotrimeric PP4 complex, PP4C-R2-R3A, on several residues on the R2 and R3A subunits, thereby reducing PP4 activity in mitosis (Voss et al., 2013). This reduction in PP4 activity during mitosis is known to be essential for keeping γ-tubulin at the centrosomes phosphorylated, which enhances the formation of the mitotic spindle.

## Phosphatases fighting back–PPP driven regulation of kinase and phosphatase activities

PPPs are not only active throughout mitosis to counteract kinases on shared substrates and regulate substrate phosphorylation site occupancy, but they also directly regulate kinase activities. One common mechanism is by modulation of protein kinase activation loop phosphorylation occupancy (Figure 2). For many protein kinases, phosphorylation site occupancy of the activation loop correlates with their activity, constituting an effective mechanism to impose phosphatase control on kinase activity. For instance, PP1 in complex with different regulatory subunits dephosphorylates AURKB, Plk1, Mps1, and Gwl (Yamashiro et al., 2008; Posch et al., 2010; Moura et al., 2017; Ren et al., 2017). PP6 does the same with AURKA (Figure 2).

For full reactivation of PP2A-B55, Gwl phosphorylation of ENSA and ARPP19 must cease. After the decline of Cdk1/cyclin B activity, PP1 auto-dephosphorylates Thr-320 resulting in its reactivation (Wu et al., 2009). Once reactivated, PP1 catalyzes the inactivation of Gwl in anaphase (Figure 2). This is achieved through the action of PP1γ in complex with the regulatory subunit 3B (PPP1R3B) (Ren et al., 2017). PP1 dephosphorylates several phosphorylation sites on Gwl, including Ser-883 in the activation loop, leading to a reduction in Gwl activity and reactivation of PP2A-B55 (Ma et al., 2016; Rogers et al., 2016; Ren et al., 2017). Interestingly, another phosphatase FCP1 was also implicated in the inactivation of Gwl (Della Monica et al., 2015) or dephosphorylation of ENSA (Hegarat et al., 2014), suggesting more complex regulatory interactions between kinases and phosphatases. In fission yeast, the PP1-dependent reactivation of PP2A-B55 starts a relay resulting in the reactivation of PP2A-B56 (Grallert et al., 2015). PP2A-B55 dephosphorylates Ser-378 in the PP1 docking motifs on the B56 regulatory subunit of PP2A. Phosphorylation of Ser-378 is carried out by Plk1 and only upon reduction of Plk1 activity in telophase, PP2A-B55 can sufficiently dephosphorylate B56 to promote PP1 binding and PP2A-B56 activation (Grallert et al., 2015).

PP1 is also implicated in the regulation of AURKB activity. In complex with Sds22 (PPP1R7), PP1 reverses AURKB activation loop phosphorylation (Thr-232 in human cells) at kinetochores (Posch et al., 2010). Sds22 depletion, likely through hyperactivation of AURKB, leads to defects in kinetochore-microtubule interactions and an increase in inter-kinetochore distance. A heteromeric complex of PP1 and Mypt1 (PPP1R12A) counteracts AURKA phosphorylation of the activation loop of Plk1, regulating Plk1 activity during mitosis (Macurek et al., 2008; Seki et al., 2008; Yamashiro et al., 2008). Finally, it was recently shown in *Drosophila* that PP1 can dephosphorylate the activation loop site of the checkpoint kinase Mps1, thereby inactivating it upon the proper attachment of kinetochores (Moura et al., 2017). This is a crucial step for silencing of the spindle assembly checkpoint (SAC) and activation of the APC/Cdc20 complex to drive anaphase progression.

PP6 dephosphorylates the activation loop phosphosite of AURKA (Figure 2; Zeng et al., 2010). AURKA is activated by autophosphorylation of its activation loop (Thr-288 in human cells) and binding of the activator Tpx2 (Eyers et al., 2003; Zorba et al., 2014). Hyperactive AURKA results in defects in chromosome segregation and spindle assembly. Depletion of PP6 has also been shown to cause spindle timing, spindle positioning, and chromosome segregation defects. This function of PP6 seems to be conserved in other species, including *Drosophila melanogaster* and *Caenorhabditis elegans*, suggesting conservation of its role in regulating AURKA activity (Chen et al., 2007; Afshar et al., 2010).

PP4 is known to regulate Cdk1/cyclin B activity through the regulation of its partner cyclin B (Figure 2). In mitosis, cyclin B is phosphorylated by Cdk1 on Ser-126 (in human cells) at centrosomes (Jackman et al., 2003). In G2, centrosome-localized PP4 dephosphorylates cyclin B and suppresses Cdk1 activity. Upon entry into mitosis, PP4 delocalizes from centrosomes promoting Cdk1/cyclin B activation. Prolonged localization of PP4 at centrosomes prevents Cdk1/cyclin B activation and mitotic progression.

## The impact of the phosphorylation site on PPP substrate dephosphorylation

We have made great advances in understanding how protein kinases recognize their substrates through protein-protein interactions, scaffolding proteins, and linear sequence motifs surrounding the phosphorylatable amino acid. But how do PPPs recognize substrates? Are these general themes of kinase substrate recognition conserved in PPPs? In recent years, a reawakening in research interest in PPPs has led to the discovery of active site preferences and linear sequence motifs around the phosphorylated amino acids and elsewhere as PPP-substrate recognition themes.

Proteins can be viewed as assemblies of structured domains connected by intrinsically disordered regions. These disordered regions frequently contain linear sequence motifs that are implicated in establishing protein-protein interactions and are often sites of post-translational modifications, including phosphorylation. Serine/threonine protein kinases are classified as basophilic, acidophilic, or proline-directed based on their preference to phosphorylate specific amino acids (Ser/Thr/Tyr) surrounded by linear sequence motifs containing either basic amino acids or acidic amino acids or proline (Manning et al., 2002; Miller et al., 2008; Kettenbach et al., 2012). In addition, some protein kinases have been shown to exhibit a preference to phosphorylate either serine or threonine residues (Kettenbach et al., 2012). Excitingly, similar observations for the preferences of PPPs for specific phosphorylation site linear motifs and phosphorylated residues have recently been made.

A series of recent studies demonstrated that during mitotic exit phospho-threonine followed by a proline (pTP) were more rapidly dephosphorylated than pSP sites and that this is due to the action of the phosphatase PP2A-B55 (McCloy et al., 2015; Cundell et al., 2016; Godfrey et al., 2017). Interestingly, the preference of PP2A-B55 for threonine over serine amino acids is conserved from yeast to humans (Cundell et al., 2016; Godfrey et al., 2017). The phospho-amino acid preference of PP2A-B55 can potentially be explained by a more energetically favorable fit of threonine over serine in the active center of the PP2A catalytic subunit (Rogers et al., 2016). PP2A-B55 does dephosphorylate phospho-serines. However, this occurs with slower kinetics and appears to require that the phospho-serine is part of linear sequence motif that contains an upstream aromatic or bulky hydrophobic residue (Cundell et al., 2016). In addition, phosphoproteomic analyses of candidate PP2A-B55 substrates revealed a correlation between the net charge of amino acids surrounding the phosphorylation site and dephosphorylation kinetics. (Cundell et al., 2016). The more basic a substrate was, the faster it was dephosphorylated. Preferred substrates of PP2A-B55 have basic amino acids downstream directly next to the phosphorylation site, as well as 10–15 amino acids upstream of it, generating a bipartite polybasic recognition motif. Furthermore, acidic residues were underrepresented downstream of the phosphorylation sites. PP2A-B55 identifies these basic linear motif elements in substrates through acidic patches on the surface of B55 regulatory subunit (Cundell et al., 2016). These preferences also provide an explanation for the observation that ENSA is an “unfair” competitive substrate/inhibitor with a high affinity for PP2A-B55 and slow dephosphorylation kinetics (Williams et al., 2014). Besides being an inhibitor, phosphorylated ENSA is also a substrate of PP2A-B55. The Gwl phosphorylation site in ENSA is a serine that is surrounded by a bipartite polybasic recognition motif which likely contributes to high affinity binding of ENSA to PP2A-B55. However, dephosphorylation of serine occurs with a slower kinetics, increasing ENSA residence time in the active site of PP2A-B55.

Both PP1 and PP2A have been found to have a preference for dephosphorylating basic and proline-directed phosphorylation site sequences (Wurzenberger and Gerlich, 2011; Rogers et al., 2016). This has been attributed to acidic residues on the surface of both enzymes which promotes binding to basophilic patches on their substrates (Egloff et al., 1995). However, there are several kinases that preferentially phosphorylate sites surrounded by upstream or downstream acidic amino acids such as Plk1 and CK2 (Miller et al., 2008; Kettenbach et al., 2012). We have recently shown that PP6 dephosphorylates acidic phosphorylation site sequences in mitosis and opposes CK2 phosphorylation (Rusin et al., 2015). One example of an acidic phosphorylation site sequence can be found in the condensin I complex, which is specifically dephosphorylated by PP6 but not by PP2A-B55.

**Figure 3:**
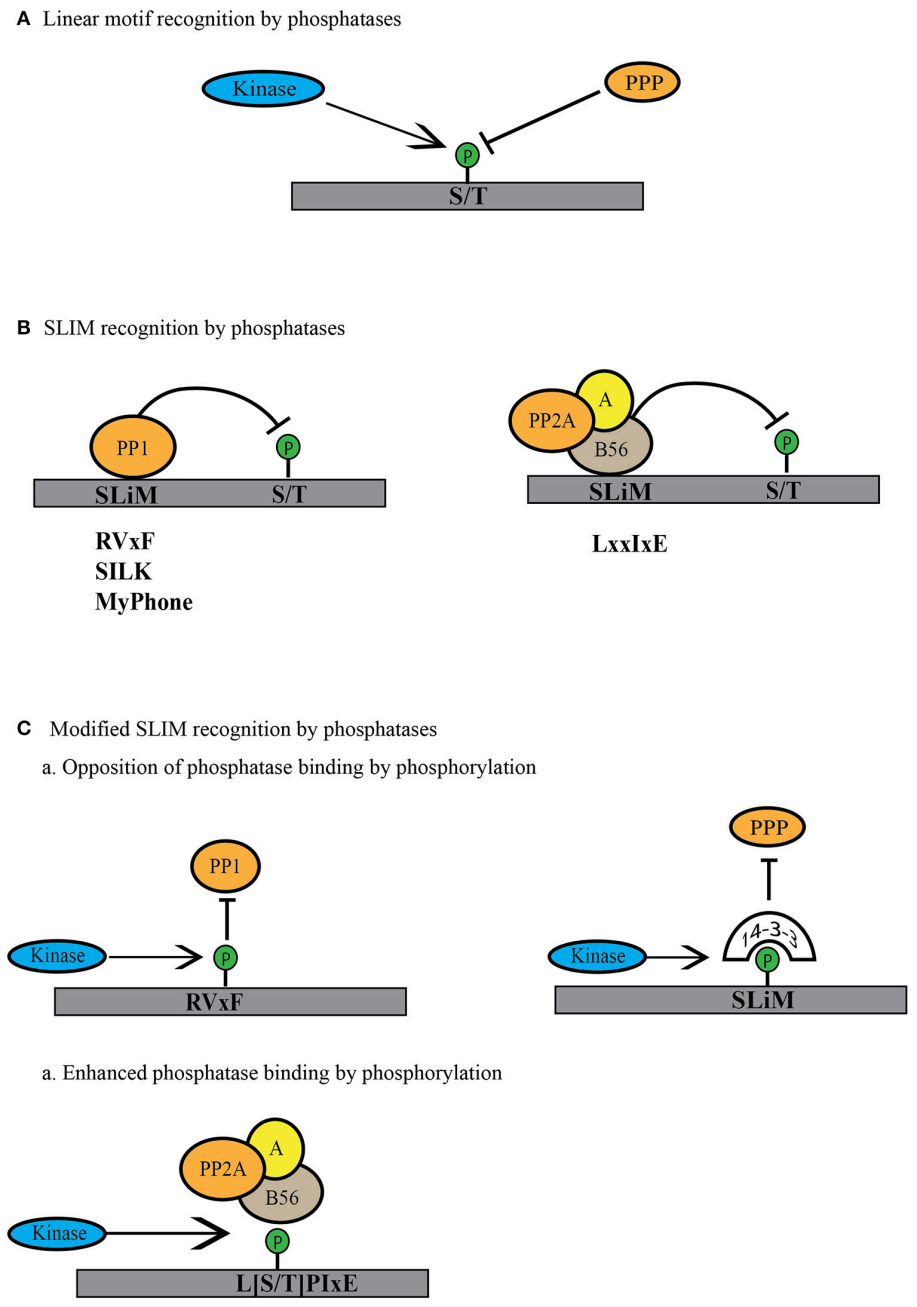
Modes of linear motif recognition by protein phosphatases. **(A)** Linear motifs can dictate the binding preferences of PPP family phosphatases. This can include a preference for the phosphosite (serine or threonine) or a preference for the residues surrounding the phosphosite (basic, acidic or proline-directed). **(B)** PPPs recognize short linear motifs (SLiMs) in regulatory proteins or substrates. PPP binding via the SLiMs helps them recognize and bind the regulatory proteins or the substrates to dephosphorylate them. PP1 is known to bind through RVxF, SILK and MyPhone motifs to its regulatory proteins (shown in gray). B56 regulatory subunit of PP2A binds through LxxIxE motifs to its substrates (shown in gray). **(C)** Phosphatase activity can be regulated by modulation of the SLiM motif. Phosphorylation within or near the SliM sequence can lead to decreased phosphatase binding either by direct blocking in the case of PP1 or indirectly by binding to phospho-binding 14-3-3 proteins to block the site of interaction. In case of PP2A-B56 SLiM, this phosphorylation can enhance phosphatase activity toward substrate by increasing the affinity for phosphorylated SLiM.

The active sites of human PPPs (PP1α-γ, PP2A, PP2B, PP4, PP5, PP6, and PP7) are 100% identical (Shi, 2009). How can the reported substrate preferences of PPPs be explained? How is it that PP1 and PP2A favor basic and proline-directed phosphorylation site sequences, PP2A-B55 dephosphorylates either threonines or serines only in combination with aromatic or bulky hydrophobic residues, and PP6 acidic sites (Figure 3A)? While the active site of the PPP catalytic subunit is 100% conserved, its conformation can be differentially modulated by substrates as well as PPP regulatory and scaffolding subunit binding. These mechanisms remain to be explored for each PPP holoenzyme and will help us to match protein phosphatase and kinase based on their shared substrate preferences.

## The role of short linear motifs and their regulation of PPP holoenzyme assembly and substrate recognition

Besides directly influencing the biochemical characteristics of the phosphorylation sites, short linear motifs (SLiMs) also contribute to the assembly of PPP holoenzymes and the recognition of substrates. SLiMs are often located in intrinsically disordered regions of proteins and the sequence composition of the SLiM regulates protein-protein interaction and binding affinity. SLiM-mediated interactions promote PPP holoenzyme assembly, substrate specificity, phosphatase activity, and subcellular targeting. The use of SLiMs as a primary mechanism for catalytic and regulatory subunit assembly was first discovered for PP1 and PP2B (Calcineurin, PP3) (in detail reviewed in Roy and Cyert, 2009; Bollen et al., 2010; Heroes et al., 2013; Figure 3B). Since then, it has become clear that SliMs are not only important for establishing interactions between catalytic and regulatory subunits, but also contribute to the regulation of these interactions. SLiMs can be post-translationally modified and these modifications strongly impact the binding affinity and targeting of phosphatases (Figure 3C). Phosphorylation of SLiMs is an effective mechanism for regulatory interventions of PPP holoenzyme assembly and substrate recognition and establishes feedbacks that enable rapid switching of phosphatase and kinase activities in mitosis. Here, we discuss the role of PP1 and PP2A SLiMs in the regulation of PPP activity in the context of mitosis.

Approximately 90% of PP1 regulatory subunits contain a RVxF motif ([K/R][K/R][V/I]{P}[F/W]; {} excluded amino acid) through which they associate with the catalytic subunit (Bollen et al., 2010; Heroes et al., 2013). Along with the RVxF motif, PP1 regulatory proteins often contain additional short linear motifs like S/GILK and MyPhone (myosin phosphatase N-terminal element), which provide sites of secondary interaction with the catalytic subunit (Heroes et al., 2013; Figure 3B). The SliM-dependent association of the regulatory subunit with the PP1 catalytic subunit generates a holoenzyme that then dephosphorylates specific substrates, including in some cases the regulatory subunit itself. A large number of SLiM-dependent interactions regulate the activity of PP1 during mitosis. At the onset of anaphase, PP1 is recruited to chromosomes through an RVxF-dependent interaction with Repo-Man (recruits PP1 onto mitotic chromatin in anaphase, also called CDCA2) to dephosphorylate histone H3 at Thr-3 (Trinkle-Mulcahy et al., 2006; Qian et al., 2011, 2013a). Histone H3 Thr-3 phosphorylation is essential for AURKB recruitment to chromosomes (Wang et al., 2010). In prophase, AURKB localizes to chromosomes, but is restricted to centromeres in the later stages of mitosis through the dephosphorylation of histone H3 Thr-3 by Repo-Man-PP1 complex (Qian et al., 2011). At centromeres and kinetochores, PP1 levels are tightly controlled; too much or too little results in mitotic defects. This is achieved through AURKB itself, the activity of which follows a gradient, being the highest at centromeres and tapering toward chromosome arms. AURKB phosphorylates basic consensus motifs (Kettenbach et al., 2011, 2012); thus, if the “x” in the PP1 SLiM RVxF is a serine or threonine amino acid, it is a consensus site for AURKB and can be phosphorylated. For instance, AURKB phosphorylates Repo-man on Ser-893, thereby inhibiting its binding to histones (Qian et al., 2013a). PP1 is also recruited to kinetochores through RVxF-dependent interactions with kinetochore null 1 (KNL1), kinesin-like protein 18A (Kif18A) (Posch et al., 2010; Rodrigues et al., 2015), and centromere-associated protein E (CENP-E) (Kim et al., 2010; Liu et al., 2010; De Wever et al., 2014). In the case of KNL1, AURKB directly phosphorylates Ser-60 within the RVxF motif and Ser-24 within the SILK motif, abrogating the recruitment of the PP1 catalytic subunit (Liu et al., 2010). For Repo-Man and KNL1, AURKB phosphorylation is opposed by another PPP, PP2A-B56 (Nijenhuis et al., 2014; Qian et al., 2015). PP2A-B56 is important for the appropriate balance of both PP1 and AURKB activities as cells progress from prophase to metaphase, when the chromosomes attach to the mitotic spindle through kinetochore-microtubule interactions and align at the metaphase plate. These processes are monitored by the spindle assembly checkpoint (SAC), a surveillance mechanism that ensures correct microtubule-kinetochore attachment and inter-kinetochore tension (Rosenberg et al., 2011; Moura et al., 2017). Until chromosome congression, the SAC generates a “wait” signal to block metaphase to anaphase transition and ensures proper chromosome alignment before sister chromatid separation (Waters et al., 1998; Skoufias et al., 2001). Part of the “wait” signaling is the recruitment of PP2A-B56 complex to kinetochores through its binding to the SAC component BubR1 (Taylor et al., 1998; Suijkerbuijk et al., 2012; Kruse et al., 2013). After proper biorientation is achieved, PP2A-B56 dephosphorylates Ser-893 on Repo-Man and Ser-60 and Ser-24 on KNL1 promoting PP1 recruitment to chromosomes and kinetochores, respectively (Qian et al., 2013a, 2015; Nijenhuis et al., 2014). Upon anaphase onset, AURKB translocates to the midzone and cyclin B is degraded, resulting in increased recruitment of Repo-Man-PP1 to chromosomes and centromeres and promotion of nuclear envelope reassembly (Vagnarelli et al., 2011).

The SLiM for PP2A-B56 was only recently identified in the SAC protein BubR1 (Suijkerbuijk et al., 2012; Kruse et al., 2013; Qian et al., 2013a; Xu et al., 2013). Using mass spectrometry-based proteomics as well as structural approaches, it was shown that a LxIxxE or an expanded [L/C/V/M/I/F][S/T]P[I/L/V/M]xE sequence represents a general, highly conserved consensus sequence for PP2A-B56 interactions (Figure 3B; Hertz et al., 2016; Wang et al., 2016). Interestingly, this mode of protein-protein interaction is not conserved in PP2A-B55 (Hertz et al., 2016). The B56 SLiM can also be modulated by phosphorylation (Hertz et al., 2016; Wang et al., 2016). However, while phosphorylation of RVxF motif decreases PP1 affinity, phosphorylation of the B56 SliMs increases its affinity for PP2A (Figure 3C). For instance, in case of Repo-Man, direct phosphorylation of the RVxF motif by AURKB or phosphorylation of [S/T]P sites adjacent to RVxF motif by Cdk1/cyclin B decrease binding of PP1 to Repo-Man (Qian et al., 2013a, 2015). Conversely, Cdk1/cyclin B phosphorylation of the PP2A-B56 SLiM motif (LpSPIxE) increases PP2A recruitment to Repo-Man (Qian et al., 2015).

Another possible mode of regulation of SLiMs arises from proteins containing phospho-binding domains. Like protein kinases and phosphatases, phospho-binding domains display serine/threonine or tyrosine specificity (Reinhardt and Yaffe, 2013). Serine/threonine phospho-binding domains include 14-3-3 proteins, WW domains, WD40 repeats, FHA domains, BRCT domains, and Polo-box domains and have been implicated in the regulation of cell cycle progression (Reinhardt and Yaffe, 2013). Proteins containing these domains bind to phosphorylated amino acids and regulate their stability, subcellular localization, activity, and protein-protein interactions. Thus, it is possible that binding of phospho-binding domain containing proteins to phosphorylated SLiM motifs could be an additional regulatory mechanism to control PPP holoenzyme assembly (Figure 3C). While not described for SLiM phosphorylation yet, the binding of 14-3-3 proteins plays an important role in the regulation of Cdc25, a PTP phosphatase. Activation of Cdk1/cyclin B is governed by multiple positive and negative feedback loops (Morgan, 2007). Cdc25 contributes to the activation of Cdk1/Cyclin B by dephosphorylating crucial inhibitory phosphorylation sites on Cdk1, Thr-14, and Tyr-15 (Honda et al., 1993). Cdc25 activity is also controlled by activating and inhibitory phosphorylations and 14-3-3 proteins bind to the inhibitory phosphorylation sites on Cdc25, further reducing Cdc25 activity, and localizing Cdc25 in the cytoplasm (Kumagai et al., 1998; Graves et al., 2001). Activation of Cdc25 requires, at least in part, the removal of 14-3-3 through dephosphorylation of the phospho-binding site. This is achieved by PP1 and PP2A-B56. PP1 dephosphorylates the 14-3-3 binding sites, while PP2A-B56 dephosphorylates additional sites that contribute to 14-3-3 release (Margolis et al., 2006a,b).

## Perspective

Protein kinases and phosphatases are highly specific enzymes with tightly regulated activities. While protein kinases have been in the spotlight of phosphorylation research for the past four decades, protein phosphatases are now taking center stage. As we learn more about the regulatory mechanism(s) governing both classes of enzymes, it is becoming clear that protein kinases and phosphatases not only counteract each other on their shared substrates but also counteract each other directly as well as indirectly through elaborate feedforward and feedback loops. Identification of regulatory mechanisms governing the activity of distinct PPP holoenzymes including phospho-amino acid (Ser vs. Thr) and phosphorylation site motif (acidophilic vs. basophilic) preferences have greatly increased our knowledge of dephosphorylation signaling and demonstrate the importance of investigating PPP holoenzyme specific substrate preferences *in vitro* and *in vivo*.

The identification of SLiM mediated subunit-subunit and subunit-substrate interaction mechanisms has greatly enhanced our ability to predict PPP regulatory subunits and substrates and build PPP-based networks. SLiMs provide an entry point for specifically targeting PPPs to discover their biological functions and substrates as well as roles in diseases. Phosphorylation signaling is frequently disrupted or deregulated in many types of cancer. Until now, the focus for therapeutic strategies has been on protein kinases. However, the inevitable emergence of kinase inhibitor resistance requires new therapeutic approaches and targeting regulatory PPP subunits and their interactions with catalytic subunits may prove to be a viable strategy. PPP inhibition activates the spindle assembly checkpoint and blocks mitotic exit, ultimately leading to mitotic catastrophe and cell death, a strategy often applied in cancer therapy. While global inhibition of PPP activity impacts all cells, targeting specific PPP holoenzymes via the SLiM motif could be tailored to signaling pathways frequently deregulated in cancer. For instance, selective inhibition of PP1 in mitosis has been shown to cause impaired tumor growth (Winkler et al., 2015). PP2A has been characterized as a tumor suppressor gene and activation of PP2A inhibits tumor growth (Sangodkar et al., 2016, 2017), while inhibition of PP4 and PP6 induces cancer cell death and sensitizes to radiation therapy (Shen et al., 2011; Theobald et al., 2013).

Another important area of investigation in PPP biology is the identification of substrates of specific PPP holoenzymes in order to connect kinases and PPPs on their shared substrates. Quantitative mass spectrometry-based proteomic and phosphoprotoemic approaches combined with small molecule kinase inhibitors, genetically-engineered ATP-analog sensitive kinases, substrate trapping mutants, proximity-labeling and crosslinking approaches have identified a plethora of kinases substrates. Using similar approaches when applicable and available, as well as inducible degron strategy and SLiM targeting peptides to identify the phosphatase substrateswill fill the gaps in our understanding of phosphorylation signaling networks. In addition, identification of upstream signaling inputs regulating PPP holoenzyme assembly, activity and substrate targeting will be important to dissect the complex regulatory behaviors.

## Author contributions

IN and AK wrote the manuscript and made a direct, intellectual contribution to the manuscript.

### Conflict of interest statement

The authors declare that the research was conducted in the absence of any commercial or financial relationships that could be construed as a potential conflict of interest.
